# Development of an efficient vector system for gene knock-out and near in-*cis* gene complementation in the sugarcane smut fungus

**DOI:** 10.1038/s41598-017-03233-7

**Published:** 2017-06-08

**Authors:** Shan Lu, Xiaorui Shen, Baoshan Chen

**Affiliations:** 1 State Key Laboratory of Conservation and Utilization of Subtropical Agro-Bioresources, Nanning, 530004 China; 2 College of Life Science and Technology, Guangxi University, Nanning, 530004 China

## Abstract

*Sporisorium scitamineum* is the causative agent responsible for smut disease of sugarcane worldwide. However, lack of efficient gene manipulation system makes this fungus much behind the type model of the smut fungi in molecular biology. Here, we report the development of a CRISPR/Cas9 and T-DNA based dual vector system that allowed efficient knock-out or knock-in of a gene of interest in the *S. scitamineum* in a site-specific manner. By using *Mfa2*, a key player in the mating event in *S. scitamineum* as a tester gene, site-specific insertions of the introduced fragments were achieved both for *Mfa2* knockout and complementation. Of particular advantage of this system is the simplicity of selection and identification for the desired transformants by using drug resistance coupled with PCR. This system greatly facilitates the gene function study in *S. scitamineum*, and could potentially be used for other basidiomycete fungi.

## Introduction

The phytopathogenic basidiomycete fungus *Sporisorium scitamineum* is the causative agent of sugarcane smut disease worldwide^1, 2^. The diseased plants are characteristic of stunting and whip-like structure from the stalk apex composed of a mixture of plant tissues and fungal basidiospores at the late stage of infection^3^, resulting in severe economic losses. *S. scitamineum* exhibits three different phases of lifestyles: nonpathogenic haploid yeast-like sporidia, infective dikaryotic hyphae and diploid teliospores^4^. Formation of dikaryotic hyphae is initiated by the fusion of two sporidia different in the mating type^5^. After invasion of and growth in the sugarcane host, the dikaryon hyphae develop into diploid teliospores and release into the environment; meiosis occurs during teliospore germination and sporidia development^1^. In this fungus, pathogenic development is coupled with sexual development, hyphal growth and sexual cycle are crucial for disease establishment^4^.

In recent years the whole genome sequences of *S. scitamineum* strains from China, Brazil, Australia and South Africa have been determined^2, 5, 6^. Overall, the genome of *S. scitamineum* is from 19.5 to 19.99 Mb in size which contains 6550 to 6,677 predicted protein-encoding genes^2, 5, 6^. Despite of the availability of genome structure information, little is known about the function and regulation of essential genes in this fungus, mainly due to the lack of efficient gene manipulation system. In fact, as of to date, there is only one gene that has been reported to be disrupted by design in *S. scitamineum*, although *Agrobacterium*-mediated transformation (AMT) methods have been used to generate random insertion mutants. Thus, development of an efficient gene disruption and complementation system becomes an urgent need for functional genomics studies of this pathogen.

Recently, the bacterial type II clustered regularly interspaced short palindromic repeats (CRISPR) and CRISPR-associated protein (Cas9) system^7^ is swift to become a widely used technology for precision genome editing across animal^8, 9^, plant^10, 11^ and microbe including the bacteria^12^ and fungi^13–16^. The Cas9 protein functions as an endonuclease to cleave DNA target sequences specified by single guide RNA (sgRNA) molecules that introduce a double stranded break at the desired site^17^. The break was repaired by the non-homologous end joining (NHEJ) pathway and homologous recombination which may frequently result in small or large chromosomal changes^7, 18^. Unlike the cases in mammalian cells and plants, in addition to base modification of deletion or addition, insertion of larger fragment into the Cas9-cleaved site was also found in an ascomycete fungus, *Aspergillus fumigatus* ^19^.

*Agrobacterium*-mediated transformation (AMT) is a widely used gene delivery system for plants and microbes^20–23^. This system relies on the “jumping ability” of transferred DNA (T-DNA) for efficient introduction of foreign DNA fragment into the host cell through the tumor-inducing (Ti) plasmid injection process^20^. However, the released DNA fragment flanked by the T-DNA arms inserts into the host genome in a random manner^24–26^. Indeed, it is based on this feature, AMT has been used to generate mutant library for microbes^27, 28^.

In this work, we report the development of a CRISPR/Cas9 and T-DNA based dual vector system via *Agrobacterium*-mediated transformation. This method enables site-specific and efficient disruption of a gene by insertion mutagenesis and complementation of a gene of interest by targeting the selection marker of the insertion mutant for the basidiomycete fungus *S. scitamineum*. It was found that gene disruption efficiency in *S. scitamineum* ranged between 21.7% and 39.1% for the tester gene *Mfa2*, and gene complementation efficiency targeting in *Hph* sequence at efficiency greater than 74.5%. By virtue of its simplicity in selection and identification by means of drug resistance and PCR, this system represents a powerful tool for functional genomics in *S. scitamineum*.

## Results

### Strategies of designing vectors for gene knock-out and complementation

**Figure 1:**
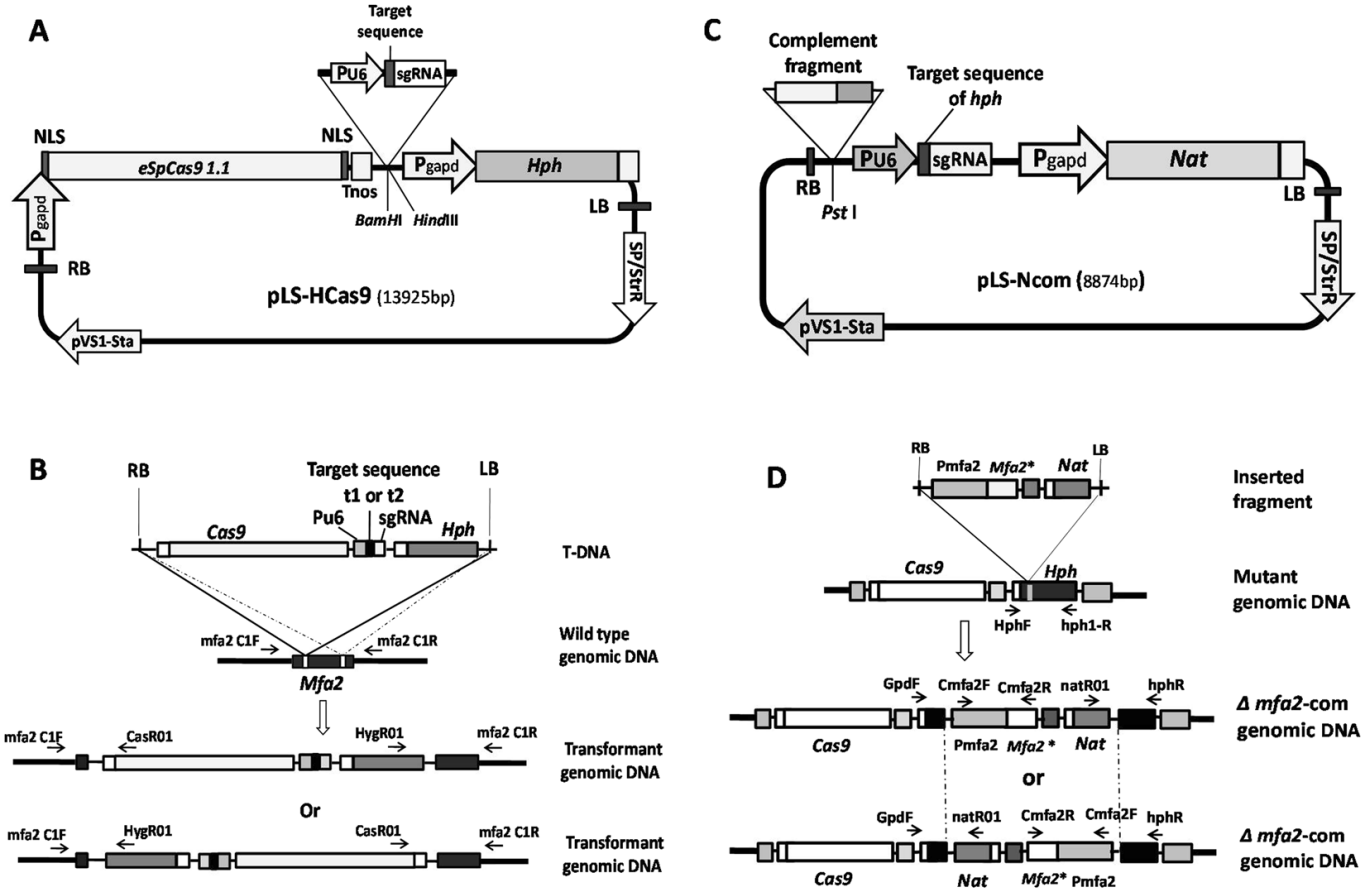
The CRISPR/Cas9 disruption and complementation system. (**A**) Structure of the pLS-HCas9 binary vector for gene disruption. (**B**) Schematic representation of the targeted gene disruption and the primers used for PCR identification. (**C**) Structure of the pLS-Ncom binary vector for gene complementation. (**D**) Schematic representation of the *Mfa2* complementation strategy and the primers used for PCR identification. *Mfa2 with target sequences modified.

We opted to adopt the *Agrobacterium*-mediated transformation system as a vehicle to deliver CRISPR/Cas9 components into the cell of *S. scitamineum*. A modified T-DNA vector for *S. scitamineum* ^4^ was used as the backbone to develop the gene disruption construct pLS-HCas9-Mfa2, in which glyceraldehydes-3-phosphate dehydrogenase (GAPDH) promoter driven *eSpCas9 1.1* ^29^ and drug selection marker *Hph* were flanked by the RB and LB arms of the T-DNA. In between the *eSpCas9 1.1* and *Hph* was the target sequence of *Mfa2* and sgRNA controlled by the *S. scitamineum* U6 promoter (Fig. 1A). It was assumed that fragment released from plasmid pLS-HCas9 via *Agrobacterium* infection would insert into the *Mfa2* target site cleaved by the Cas9, resulted in the disruption of the *Mfa2* gene. Since the integrated fragment carries a selection marker hygromycin resistance gene *Hph*, the mutant screening could be easily accomplished by the drug resistance and PCR verification with primers within the released fragment and on the target gene sequences outside the cleaved site (Fig. 1B).

The complementation construct pLS-Ncom-Mfa2 is composed of *Hph* target-sgRNA cassette, complementation gene *Mfa2*, and selection marker nourseothricin resistance gene *Nat* that were flanked by the RB and LB arms of the T-DNA in a binary vector (Fig. 1C). Upon injected into the *Mfa2* null mutant carrying *Cas9*, fragment released from plasmid pLS-Ncom-Mfa2 would insert into the *Hph* target site cleaved by the Cas9, resulted in the installation of the base-modified wild-type copy of *Mfa2* while disruption of *Hph*. The complemented transformants could be easily screened by the nourseothricin resistance positive (Nat^+^) and hygromycin resistance negative (Hph^−^), and further confirmed by PCR verification with primers within the released fragment and on the target gene sequences outside the cleaved site (Fig. 1D).

### Disruption of *Mfa2* through *Mfa2* site-specific insertion

Mfa2 was required for mating and filamentous growth in *Ustilago maydis* ^30^. The Mfa2 in *S. scitamineum* is the ortholog of *U. maydis* with identity is 53.7% (our unpublished data). Thus dysfunction of Mfa2 may be easily monitored by the phenotype of inability to get into filamentous growth after mating with a sex-opposite strain.

**Figure 2:**
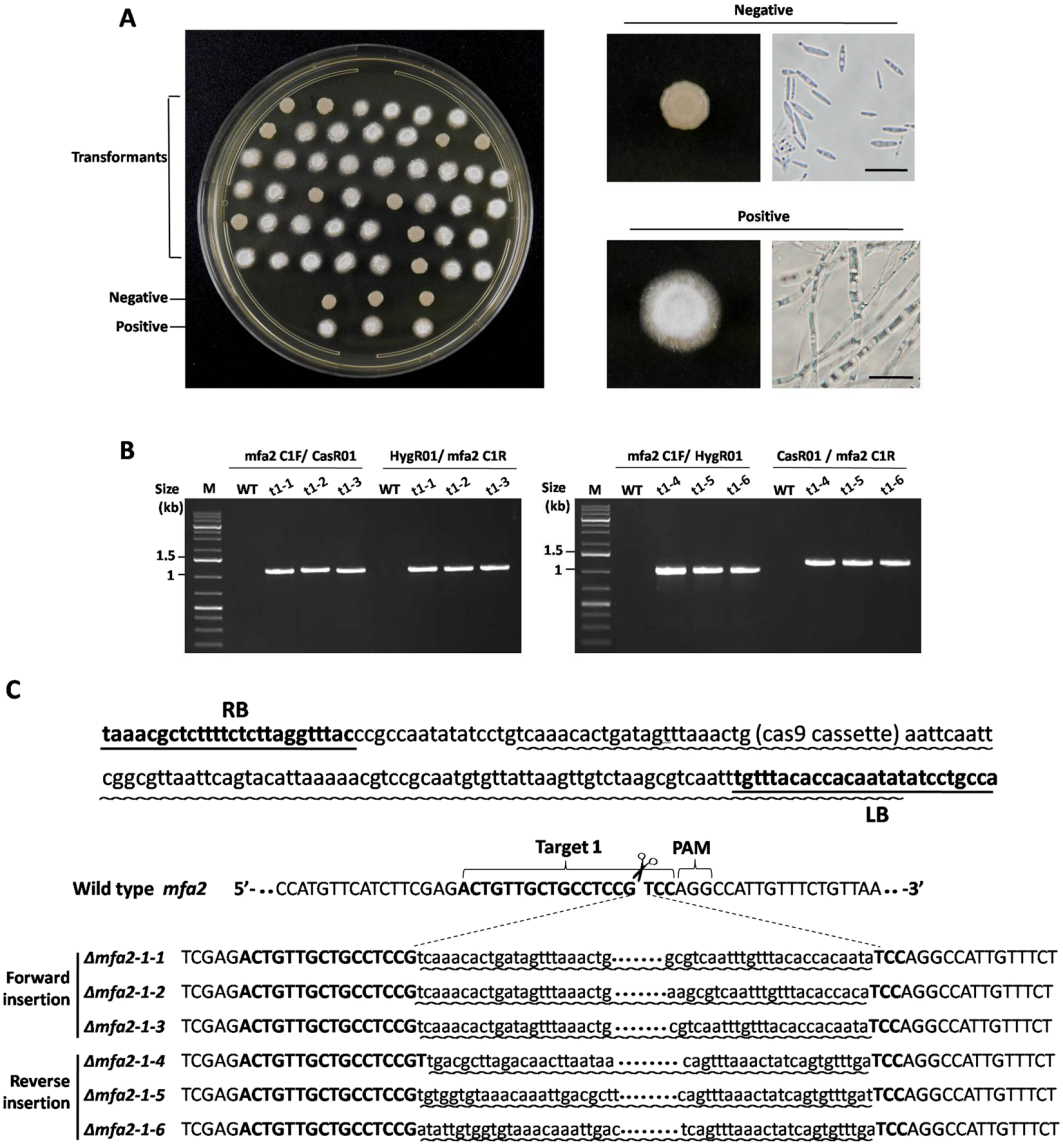
Identification and characterization of *Mfa2* targeted mutants. (**A**) Mating assays of transformants targeted at the target 1 region. At the left is the mating assay of candidate *Mfa2* mutants paring with wild type JG36. The fluffy colonies are the result of successful mating, while the dense colonies indicate a fail in mating. At the right are microscopic images of both colonies: yeast-like sporidia (mating negative) and filamentous hyphae (mating positive). Scale bars indicate 1 μm. The negative control is haploid JG35 with yeast-like colony, and the positive control is a mix of JG35/JG36 with filamentous hyphae. (**B**) PCR detection of the insert arms at the cleavage site. The left image was for the forward insertion and the right one for reverse insertion. For forward insertion, the sizes of PCR products were 1133 bp with primer pair mfa2C1F/CasR01 and 1148 bp with primer pair mfa2C1R/HygR01. For reverse insertion, the sizes of PCR products were 1030 bp by primers mfa2C1F/HygR01 and 1251 bp by primers mfa2C1R/CasR01. (**C**) Sequences of the *Mfa2* flanking the insert at target 1 locus of the transformants. Underlined are RB and LB regions. The insertion regions are marked with wavy line. As could been seen, in both forward or reverse insertions, the whole RB and a small portion of the cassette at the 5′ end were lost, but only 9–13 LB nucleotides at the far most 3′ end were lost.

To test whether CRISPR/Cas9 system works in *S. scitamineum*, *Mfa2* was chosen for gene editing. Two regions, t1 and t2, located at the 5′ and 3′ ends of the gene were selected as the targets (Supplemental Fig. S1). Introduction of pLS-HCas9-Mfa2 carrying t1 target into the smut fungus via *Agrobacterium* infection resulted in transformants resistant to hygromycin with 30–50 colonies per plate. Mating assay of the transformants with opposite sex type strain JG36 revealed that 21.7% (10 out of 46 transformants) lost the mating ability, suggesting that the gene had been dysfunctioned (Fig. 2A). PCR screening with primers up- and down-stream of *Mfa2* gene failed to specifically amplify a 700 bp *Mfa2* fragment in all the mating-defect transformants, while this fragment was present in the hygomycin-resistant transformants with normal mating function (Supplemental Fig. S2). A further PCR analysis with primers flanking RB and LB arms located at the ends of the CRISPR/Cas9 cassette revealed the CRISPR/Cas9 cassette was inserted into the *Mfa2* (Fig. 2B). Sequencing of PCR products further verified that the foreign fragment was inserted into the Cas9-cleaved site without base modification or with one or two base addition or deletion (Fig. 2C). Similar results were obtained in mutants targeted to the t2 region of *Mfa2* gene, with insertion rate of 39.1% (18 out of 46 transformants) (Supplemental Fig. S3). Southern analysis confirmed that all randomly picked transformants had single copy of *Cas9*, indicating that there was only single insertion event (Supplemental Fig. S4). The verified *Mfa2* disrupted tansformant was named *Δmfa2*.

Those transformants with hygromycin resistance but without affection to mating ability were also analyzed. No change was found in the sequences of the target regions (Supplemental Fig. S5), suggesting that the foreign fragments were inserted into the non-target regions. A further investigation to five of those transformants revealed that while the selection marker gene *hph* remained intact, the DNA fragments for CRISPR/Cas9 and sgRNA were not intact (Supplemental Fig. S6). Thus loss of CRISPR/Cas9 function resulted in the inability to cleave the *Mfa2* gene in these mutants.

### Disruption of randomly selected genes

To test if this gene disruption system works universally for other genes in *S. scitamineum*, we randomly selected 5 genes to conduct a disruption experiment. The results confirmed that the system worked well with target frequency of 12.8–38.3% (Table 1).

**Table 1:** Targeted frequency of genes by CRISPR/Cas9 and T-DNA vector system.

Gene ID	No. of Hph^+^ transformants	No. of disruptants	Targeting frequency (%)
g3949	47	6	12.8
g5775	47	14	29.8
g5019	47	18	38.3
g1621	47	10	21.2
g6375	47	17	36.1

### Complementation of *Δmfa2* through target modification and *Hph* site-specific insertion

**Figure 3:**
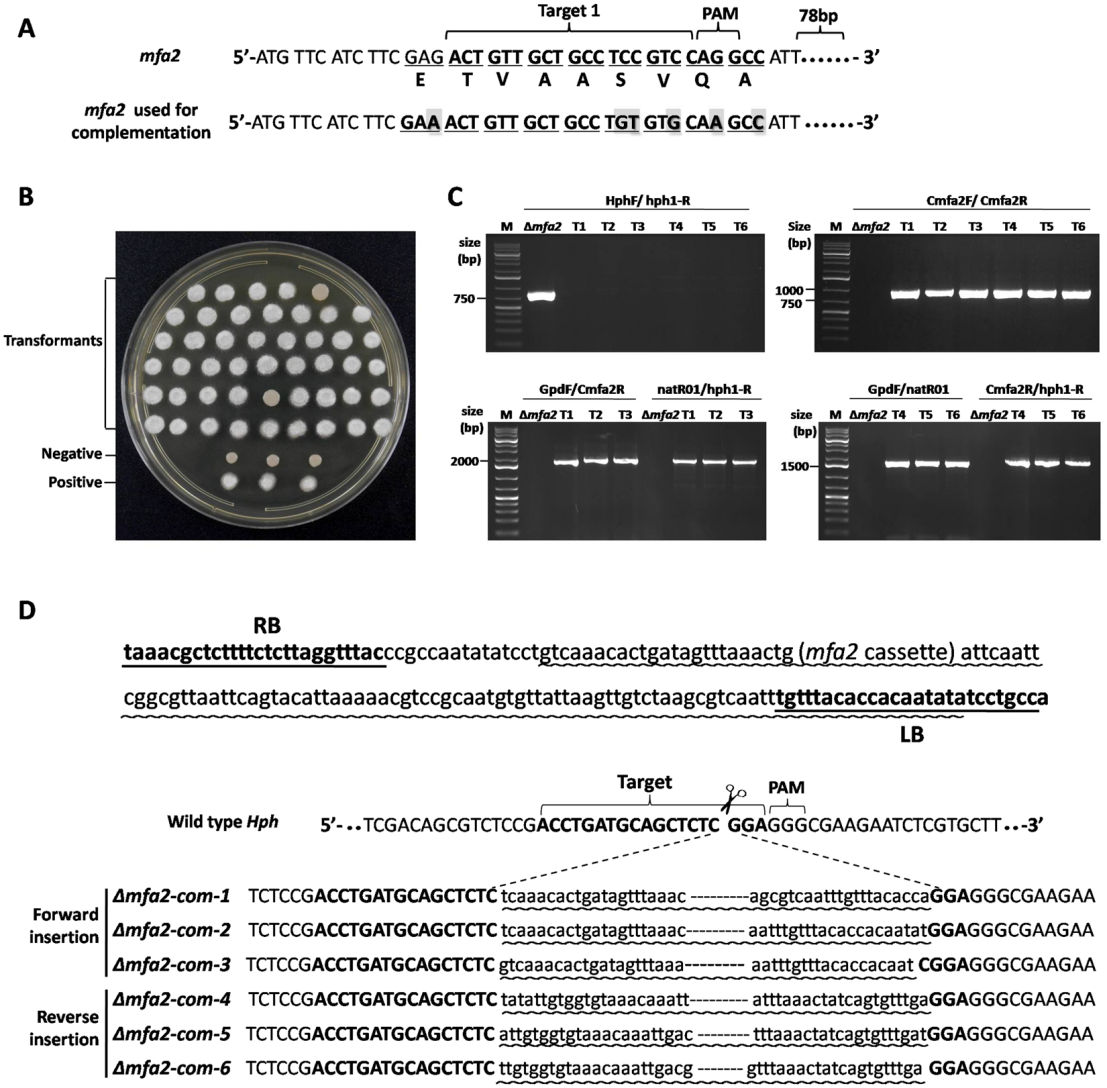
Identification and characterization of *Mfa2* complementation transformants. (**A**) Nucleotide and amino acid sequences of wild-type and base-modified *Mfa2* target locus. (**B**) Mating assays of *Mfa2* complemented transformants. The fluffy colonies are the result of successful mating, while the dense colonies indicate a fail in mating. The negative control is haploid JG35 with yeast-like colony, and the positive control is a mix of JG35/JG36 with filamentous hyphae. (**C**) PCR characterization of the complemented transformants. The up/left: detection of the *Hph* gene; the up/right: detection of the introduced *Mfa2*; the down/left: forward insertion, the sizes of PCR products were 1686 bp with primer pair GpdF/Cmfa2R and 1676 bp with primer pair natR01/hph1-R; the down/right: reverse insertion, the sizes of PCR products were 1624 bp with primer pair GpdF/natR01 and 1738 bp with primer pair Cmfa2R/hph1-R. (**D**) Sequences of the *Hph* flanking the insert at target locus of the complemented transformants. Underlined are RB and LB regions. The insertion regions are marked with wavy line. As could been seen, in both forward or reverse insertions, the whole RB and a small portion of the cassette at the 5′ end were lost, but only 8–15 LB nucleotides at the far most 3′ end were lost.

Since *Cas9* and the target sequence for *Mfa2* were already imbedded in the genome of *Δmfa2*, re-introduction of a wild-type *Mfa2* into the mutant will likely be cleaved and result in failure of complementation. To avoid this problem, the target sequence for *Mfa2* (coordinates 13–36 of *Mfa2* CDS) were subject to modification by base substitution without change of amino acid sequences (Fig. 3A). The modified *Mfa2* was cloned into pLS-Ncom to yield the complementation construct pLS-Ncom-Mfa2, which carries an *Hph*-sgRNA and *Nat* gene for nourseothricin selection (Fig. 1C). Transformation of the *Δmfa2* basidiospores with pLS-Ncom-Mfa2 by ATMT may result in trasformants resistant to nourseothricin (Nat^+^) but sensitive to hygromycin (Hph^−^). Among 380 nourseothricin-resistant transformants in 4 independent trials, 81.3% were hygromycin sensitive, indicating *Hph* had been targeted by insertion; 18.7% were hygromycin resistant, indicating off-target insertion. For those hygromycin sensitive transformants, 91.6% were restored mating ability; and for those hygromycin resistant transformants, 77.5% were restored in mating ability (Table 2). The results of PCR amplifications showed that the *Hph* gene was disrupted and the *Mfa2* gene with its prompter was inserted into the genome of the Δ*mfa2* mutant (Fig. 3C). PCR amplifications were carried out using primer sets GpdF/Cmfa2R and natR01/hph1-R or the GpdF/natR01 and Cmfa2R/hph1-R primer sets to confirm that the fragment was inserted into the target locus of *Hph* (Fig. 3C). The sequencing results of PCR products further verified that the fragment which contains the CDS of *Mfa2* gene and its promoter was inserted into the 86 nucleotides downstream of the start codon of hygromycin resistant gene which was inserted into the genome of the *S. scitamineum* when deleted the *Mfa2* gene (Fig. 3D). Transformants enable sexual mating with JG36 to develop dikaryotic filament and showing fluffy phenotype (Fig. 3B). These data demonstrated that the *Mfa2* was successfully complemented by this CRISPR/Cas9 system.

**Table 2:** Ratio of *mfa2* complementation and *Hph* disruption*.

Trail	I		II		III		IV	
	Total	Mating	Total	Mating	Total	Mating	Total	Mating
Hph^−^	80	74	80	74	78	74	71	61
Hph^+^	15	11	15	11	17	15	24	18

*Transformants were all nourseothricin resistant and numbers were transformants obtained in each trial.

## Discussion

In this paper, we report a highly efficient CRISPR/Cas9 and T-DNA based system with flexibility and precision for gene disruption and complementation in *S. scitamineum*. Unlike *U. maydis*, for which gene manipulation was achieved by transformation of basidiospores protoplast, gene knock-out or knock-in could be done by *Agrobacterium*-mediated transformation of the basidiospora directly in *S. scitamineum*. Of particular significance is that near in-*cis* gene complementation can be accomplished without interfering the function of other genes in the genome by positioning effect. To the best of our knowledge, this is the first report that a complementation gene could be re-introduced into the genome with a site-specific manner in fungi.

Homologous recombination has been widely used for gene replacement in species across the kingdoms^31–34^. To enhance the efficiency, varied modifications such as reducing the heterologous end-joining by knocking out the relevant genes, e.g. *Ku70/80* ^35–37^ and *lig4* ^38^ have been made. Unfortunately, these approaches do not work well in *S. scitamineum*, resulting in the molecular research of this fungus far-behind the model fungi. Coupling the development of the CRISPR/Cas9 and T-DNA-based vector system for site specific gene knock-out and near in-*cis* complementation with the availability of whole genome sequences information of strains of *S. scitamineum*, significant advance in *S. scitamineum* functional genomics can be expected in the near future.

As a matter of fact, T-DNA integration via homologous recombination into the host genome by aid of site-specific I-Sel nuclease cleavage of double-stranded DNA had been observed in the yeast^39^. T-DNA integration via non-homologous end joining into the host genome by aid of site-specific I-Sel or I-CeuI nuclease cleavage of double-stranded DNA was also achieved in plants^40, 41^. In this regard, the CRISPR/Cas9 and T-DNA-based vector system reported in this paper (Fig. 1A) takes advantages of a combination of site-specific cleavage of CRSPR/Cas9 system and insertion nature of *Agrobacterium* T-DNA, to greatly simplify the transformation and identification procedure.

Given the fact that the regeneration rate of *S. scitamineum* protoplast is extremely low (our unpublished data), a knock-out efficiency of 21.7–39.1% and a site-specific knock-in efficiency of 74.5% are sufficient for genetic manipulation for *S. scitamineum*. Even more important, is the use of basidospores without a need to prepare protoplasts. In this regard, the system reported in this paper represents one of the best protocols for fungi in term of genetic manipulation. Taking the merit of the precise insertion nature, single or double drug selection coupled with PCR, this system enables the identification of desired transformants in a rather simple yet reliable manner. To verify the universality of this system for gene targeting, 5 randomly selected genes from *S. scitamineum* were tested for gene inactivation. A knock-out efficiency between 12.8–38.3% with site-specific insertion was obtained (Table 1). In contrast to the primary genome editing for *Mfa2* with CRISPR/Cas9 and T-DNA-based vector (Fig. 1A) system, the rate of site-specific insertion in a secondary gene (*Hph*) targeting with CRISPR and T-DNA-based vector system (Fig. 1C) is significantly higher: about 81% (Table 2). The major difference between the two was that *Cas9* was imbedded in the fungal host cell and expressed prior to the introduction of targeting cassette in the secondary gene targeting event, while the *Cas9* was introduced into the cell together with the targeting cassette at the same time in the first event. Therefore, for a systemic investigation of gene function by gene disruption, it is better to construct an engineered strain with *Cas9* imbedded. Furthermore, since *Hph* is a foreign sequence to the fungus, complementation of any genes would be possible by targeting this region.

By counting the CRISPR/Cas9 integration events (as indicated by hygromycim resistant transformants) and the number of lost function of *Mfa2* (as indicated by failure of mating of transformants), the targeting rate was at 21.7–39.1% in transformation of *S. scitamineum* by using CRISPR/Cas9 and T-DNA based system (Figs 2 and S3). However, among the *Mfa2*-targeted transformants, CRISPR/Cas9 was all precisely integrated into the chromosome at the cleavage site of the target sequence. To our surprise, we did not find any genome modification of base indel or substitution, as those found in animal cells or plants. By close inspection of sequences at the insertion sites, we found no base modification or with one or two base addition or deletion to the chromosomal sequence at the cleavage sites (Figs 2, 3 and S3). These indicate that modification of base did happen after the DNA double helix was cleaved, and the modified ends did not join each other but rather joined with the fragment released from the transformation constructs. It was assumed that Cas9/sgRNA may form a complex with CRSPR/Cas9 cassette DNA fragment released by T-DNA signal through a partial sgRNA/sgDNA helix, and direct the CRSPR/Cas9 cassette DNA to the target site. However, the exact mechanism of extremely high rate of CRSPR/Cas9-T-DNA cassette insertion into the target site remains unknown. Furthermore, the single copy insertion (Supplemental Fig. S4) makes the CRSPR/Cas9-T-DNA system of great value for mass identification of gene function in a fungal host.

## Materials and Methods

### Synthesis of *Cas9* and construction of the CRISPR/Cas9 backbone vector

The U6 promoter fragment was amplified from *S. scitamineum* genomic DNA with primers SsPU6F/SsPU6R and then was cloned into pAtU3b^42^ plasmid to replace the AtU3b fragment by In-fusion Cloning (TaKaRa, China) to establish the sgRNA intermediate vector pSgRNA-SsU6.

The binary vector pLS-HCas9 with *Cas9* driven by the constitutive Pgapd promoter was used for genomic targeting in *S. scitamineum* through *Agrobacterium*-mediated transformation. SgDNA was fused to U6 promoter and *Cas9* was under the control of GAPD promoter. The GAPD promoter and *Cas9* gene were synthesized by Nanjing Genscript Co., Ltd. China. The cassette was cloned into the T-DNA boarded binary vector derived from pEX2^43^ with hygromycin resistance as selection marker.

### Construction of Gene Knockout Construct

Genomic sequences with 5′-GN_19_NGG-3′, where NGG is PAM, was screened and selected as candidate targets^7^. The linearized pLS-HCas9 was generated using restriction enzymes *Bam*HI and *Hin*dIII, and then was purified using DNA Fragment Purification Kit Ver.4.0 (TaKaRa, China). Target sequences were introduced into sgRNA expression cassettes by overlapping PCR. First round of PCR (20 μL) used four primers: U-F and gR-R (0.2 μM each) and two target sequence-containing chimeric primers U6T#+ and U6T#− (0.1 μM each) and PrimeSTAR MAX DNA Polymerase (TaKaRa, China) for 30 cycles (98 °C, 10 s; 58 °C, 5 s; 72 °C, 20 s). Second PCRs were set up with 0.5 μL of the PCR products and combinations of the primer pair U-Fs-BamHI/gR-R-HindIII (0.2 μM each) for In-fusion, and run for 30 cycles (98 °C, 10 s; 58 °C, 5 s; 72 °C, 30 s) to obtain a complete sgRNA expression cassette. The sequences of primers used in this study are listed in Supplementary, Table S1. The sgRNA expression cassette was purified and cloned into the binary vectors.

### Construction of Gene Complementation Construct

The sgRNA expression cassette of hygromycin phosphotransferase gene was amplified using four primers: U-F, gR-R and two target sequence-containing chimeric primers Thph+ and Thph-. The second PCRs were set up with 0.5 μL of the PCR products and combinations of the primer pair ngU-FsBamHI/NGgR-RBamHI to obtain a complete sgRNA expression cassette of *Hph*. This sgRNA expression cassette was cloned into the *Bam*HI site of binary vector pNGR1.2 which confers nourseothricin resistance, yielding binary vector pLS-Ncom.

To construct pLS-Ncom-Mfa2 for Δ*mfa2* complementation, a sequence-modified *Mfa2* was generated by substitution of the target motif including the PAM bases without changing the amino acids by PCR with the primers Cmfa2-PstIF/new-mfa2R, new-mfa2 F/Cmfa2-PstIR, separately, using genomic DNA of JG35. These two PCR products were joined together by fusion PCR and cloned into the *pst*I site of pLS-Ncom by Infusion Cloning. The inserts in all plasmids were sequenced to confirm to be error-free.

### Fungus Transformation

The binary vectors were transformed into *A. tumefaciens* strain Agl1 by electroporation^44^. Colonies carrying the vector plasmids were used for transformation of *S. scitamineum* haploid basidiospores as described previously^45^. A typical transformation experiment contained 50 μl of basidiospores with OD_600_ = 1.0 and 50 μl of *A. tumefaciens* with OD_600_ = 0.5 per plate. For selection, hygromycin B (Solaibio, China) was added to a final concentration of 200 μg/mL or nourseothricin (Solaibio, China) was added to a final concentration of 60 μg/mL. Transformants of *S. scitamineum* were verified by PCR, sequencing and subsequently used for mating assays.

### Mutation Detection

To analyze CRISPR/Cas9 mediated genome editing events in *S. scitamineum*, transformants were picked from the transformation plates, diluted and plated for single colonies on YEPS medium plates. Single colonies were grown in liquid YEPS medium and used for the isolation of genomic DNA by Plant Genomic DNA Extraction Kit (TaKaRa, China). PCR amplifications were carried out using primers flanking the designed target sites and HygR01 and CasR01. The PCR products were sequenced directly. A Southern blot analysis was performed with the DIG High Prime DNA Labeling and Detection Starter Kit II (Roche Applied Science, Indianapolis, IN), as described in Supplementary.

## Electronic supplementary material

Supplementary information
