# Supplementary material for: Development of an efficient vector system for gene knock-out and near in-cis gene complementation in the sugarcane smut fungus
Source: Sci Rep. 2017 Jun 8;7:3113. doi: 10.1038/s41598-017-03233-7 (PMC5465213; doi:10.1038/s41598-017-03233-7)
Supplement: Supplementary file 1 — Supplementary information [file 41598_2017_3233_MOESM1_ESM.pdf]

Supplementary Information

Development of an efficient vector system for gene knock-out and near in-*cis* gene complementation in the sugarcane smut fungus

Shan Lu[1,2], Xiaorui Shen[2], Baoshan Chen[1,2*]

[1]State Key Laboratory of Conservation and Utilization of Subtropical Agro-Bioresources, Nanning, 530004, China;

[2]College of Life Science and Technology, Guangxi University, Nanning, 530004, China

*Corresponding author. chenyaoj@gxu.edu.cn

S1 Methods

S1 Figures

S1 Tables

S1 Methods

Southern Blot

The number of sites into which the *Cas9* gene was integrated in *Δmfa2* mutants was analyzed by Southern blotting using the DIG High Prime DNA Labeling and Detection Starter Kit II (Roche Applied Science, Indianapolis, IN). 40 μg of genomic DNA of *Δmfa2* mutants were digested in *Hin*dIII and *Eco*RV digestion (ThermoFisher Scientific, USA) overnight at 37°C and then were electrophoresed in a 0.8% Tris-borate-EDTA (TBE) agarose gel at 100 V for 2 h. The gel was transferred onto a positively charged nylon membrane (GE, USA) overnight in 20×SSC buffer (3 M NaCl, 0.35 M sodium citrate, pH7.0). The membrane was hybridized overnight at 42°C with 25ng/ml of a 766 bp DIG probe (Roche Applied Science, Indianapolis, IN) targeted to the *Cas9* gene. The hybridized probe was immunodetected with anti-digoxigenin-AP, Fab fragments were visualized with the chemiluminescence substrate CSPD according to the manufacturer's instructions. The membrane exposes to ImageQuant LAS 500 (GE, USA) for 10 min.

S1 Figures

Target 1 PAM

***mfa2* CDS** 5’- ATGTTCATCTTCGAG**ACTGTTGCTGCCTCCGTCC**AGGCCATTGTTTCTGTTAACGAGCAAGA

Target 2 PAM

GCAGGCCCCTGTCAACGAGGGCCGTGGTCAG**CCTGCAGTCTACTGCACCG**TGGCCTAA-3’

Figure S1. Target sequences of *Mfa2* gene.

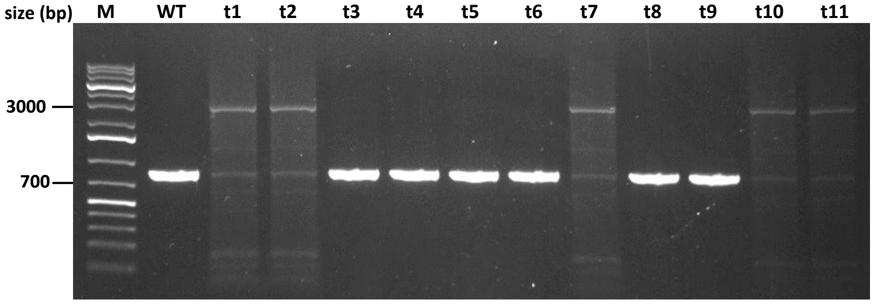

Figure S2. PCR amplification of the *Mfa2* locus of transformants. Primers used were mfa2C1F/mfa2C1R.

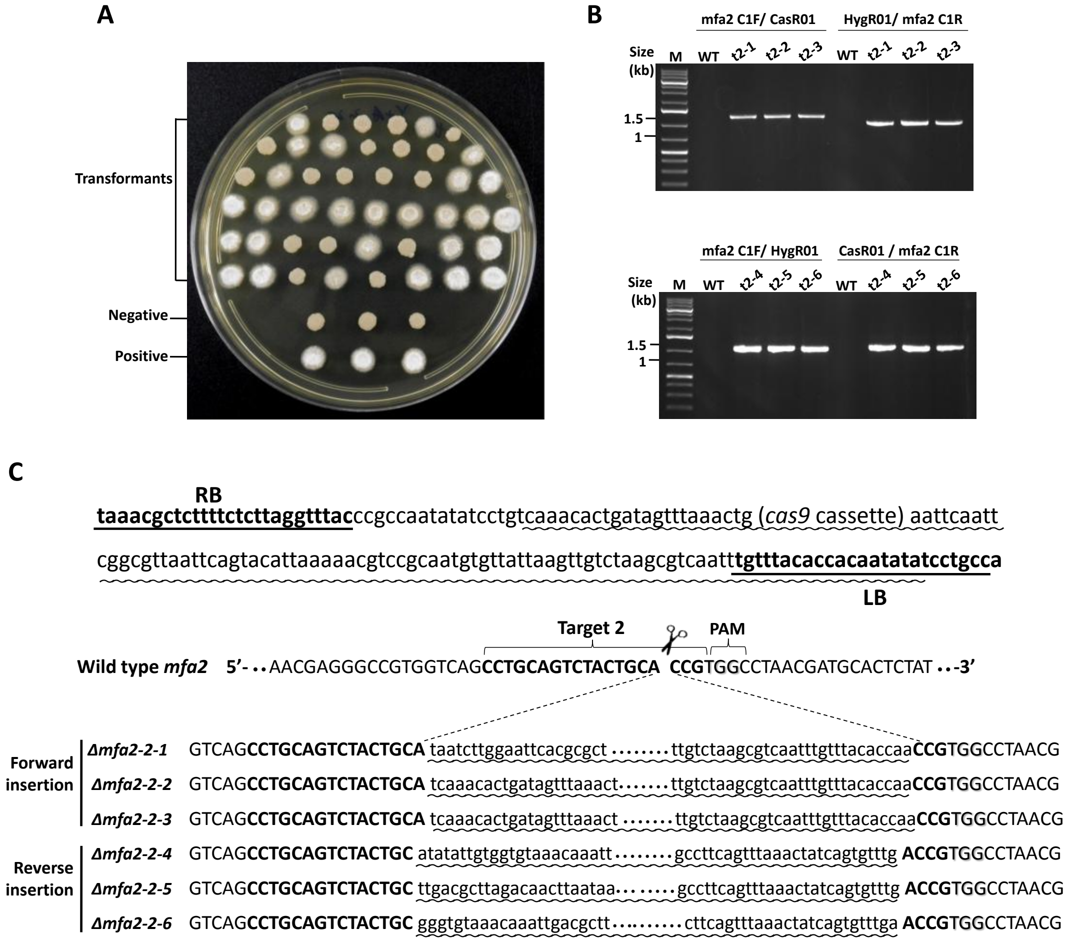

Figure S3. Identification and characterization of *Mfa2* targeted mutants. (A) Mating assays of transformants targeted at the target 2 region. The fluffy colonies are the result of successful mating, while dense colonies indicate a fail in mating. (B) PCR detection of the insert arms at the cleavage site. The left image was for the forward insertion and the right one for reverse insertion. For forward insertion, the sizes of PCR products were 1211 bp with primer pair mfa2C1F/CasR01 and 1070 bp with primer pair mfa2C1R/HygR01. For reverse insertion, the sizes of PCR products were 1108 bp by primers mfa2C1F/HygR01 and 1173 bp by primers mfa2C1R/CasR01. (C) Sequences of the *Mfa2* flanking the insert at target 2 locus of the transformants. Underlined are RB and LB regions. The insertion regions are marked with wavy line. As could been seen, in both forward or reverse insertions, the whole RB and a small portion of the cassette at the 5' end were lost, but only 7-12 LB nucleotides at the far most 3' end were lost.

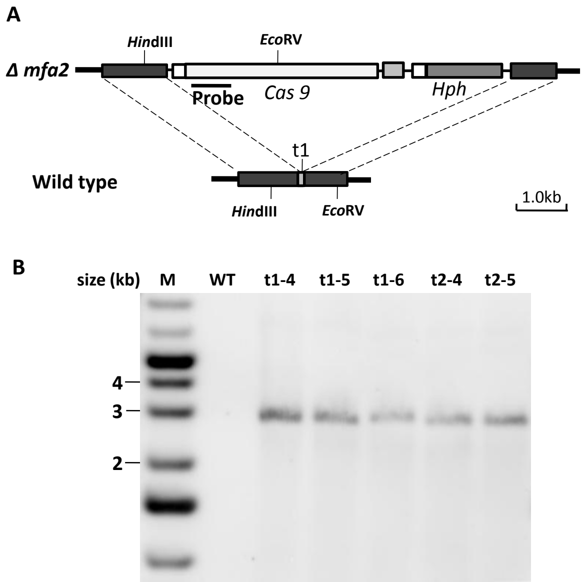

Figure S4. Southern blot analysis of the *Δmfa2* mutants. (A) Schematic representation of the location of the probe and the restriction enzymes used for DNA digestion. (B) *Δmfa2* mutants DNA were restricted with *Hin*dIII and *Eco*RV and separated in 0.8% agarose gel. After bloting, the nylon membrane was probed with labeled *Cas9* fragment. Wild-type JG35 was used as control.

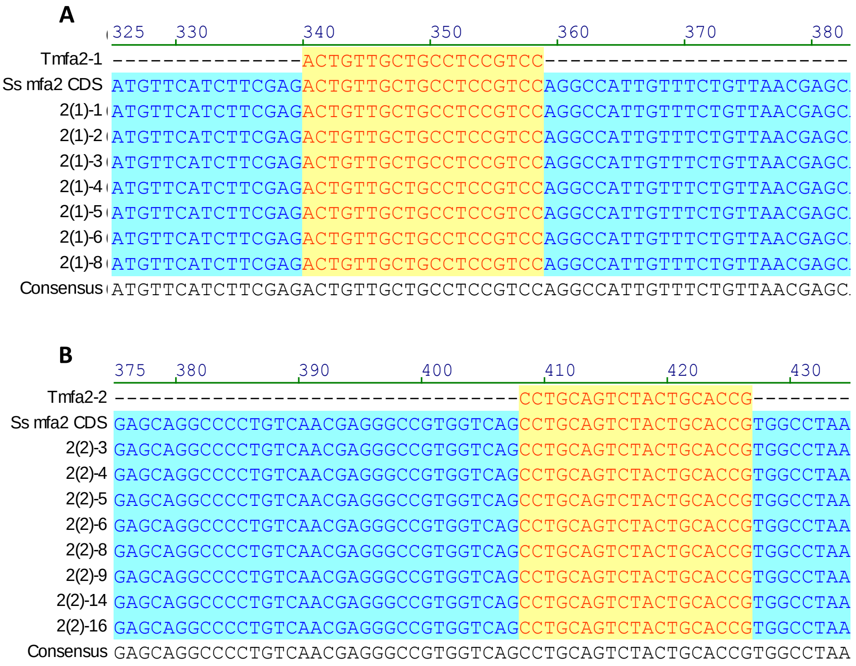

Figure S5. Sequences at target site of pLS-Hcas9-Mfa2 transformants with normal mating capability. (A) Target1. (B) Target2.

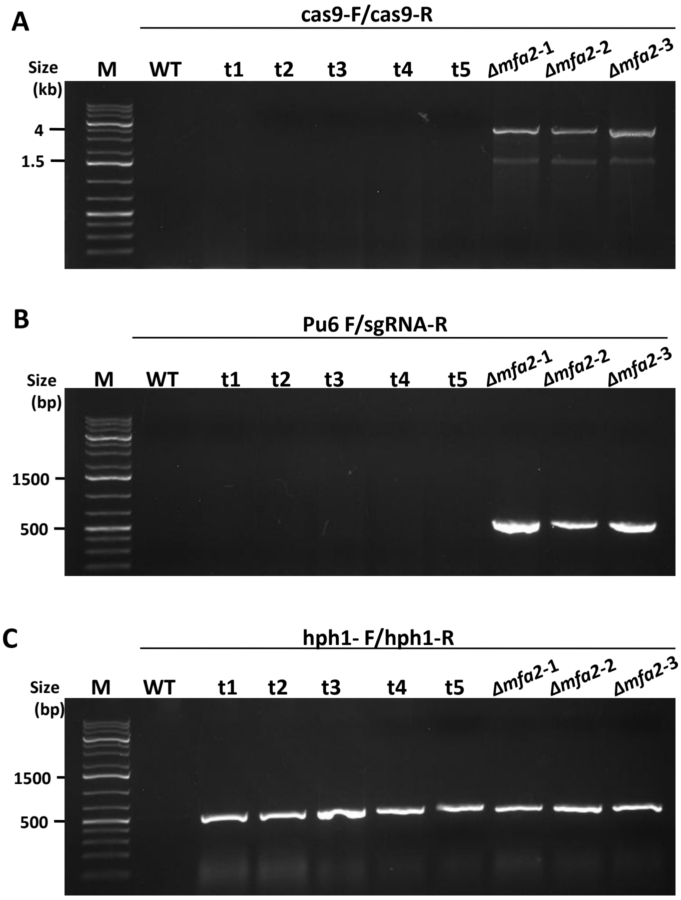

Figure S6 PCR amplification of the components of the *cas9* cassette. (A) *Cas*9 gene; (B) PU6-sgRNA; (C) *Hph* gene. The *cas9* cassettes of 5 off-target transformants with randomly integrated T-DNA delivered were not intact.

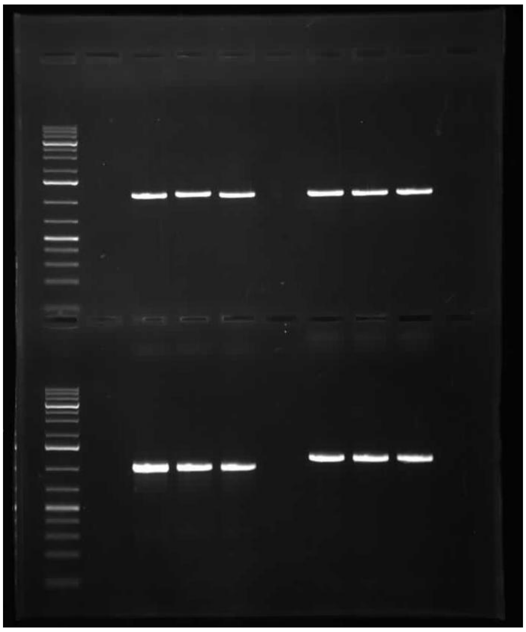

Figure S7 The full-length gel of Figure 2B.

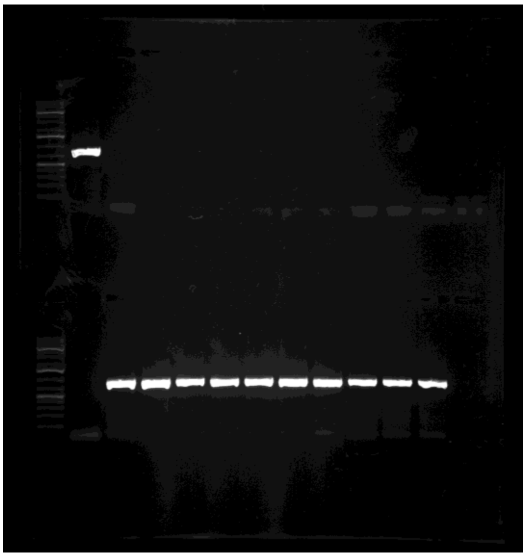

Figure S8 The full-length gel of Figure 3C (upper).

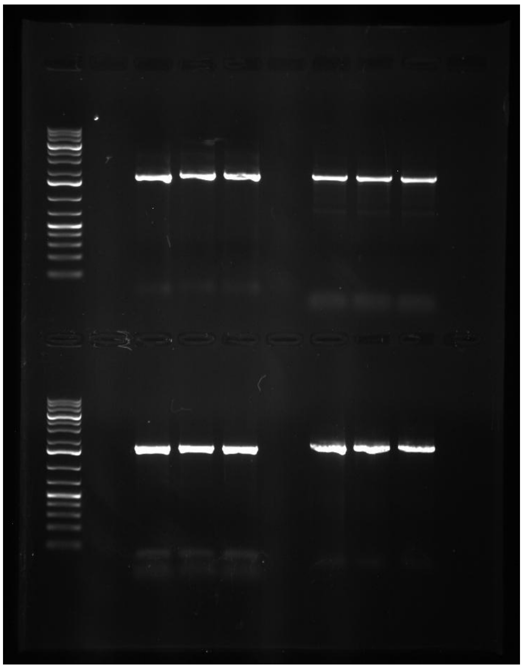

Figure S9 The full-length gel of Figure 3C (lower).

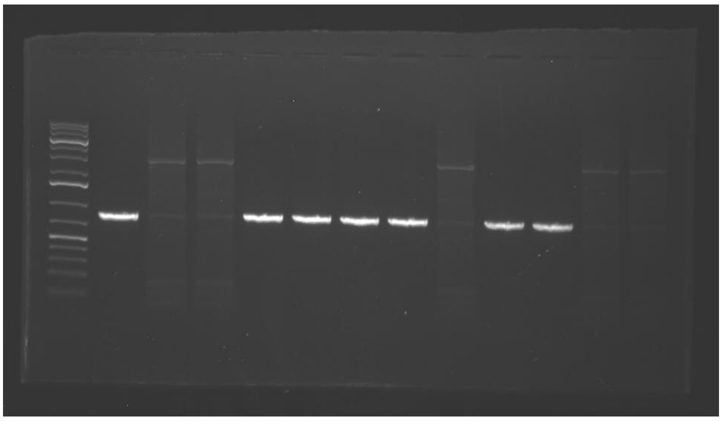

Figure S10 The full-length gel of Figure S2.

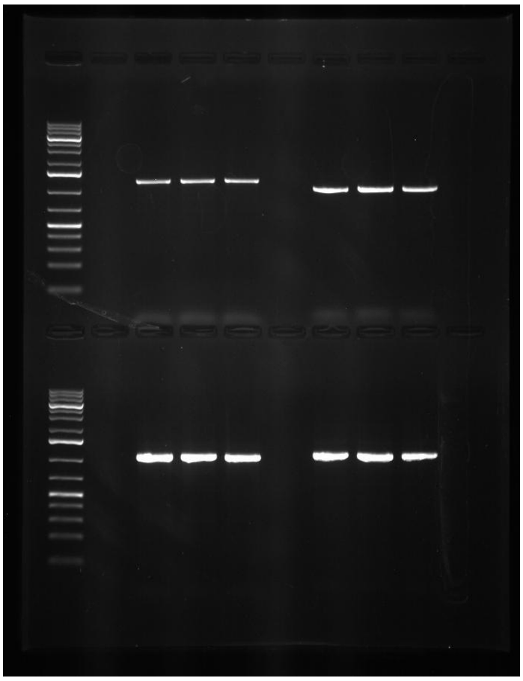

Figure S11 The full-length gel of Figure S3B.

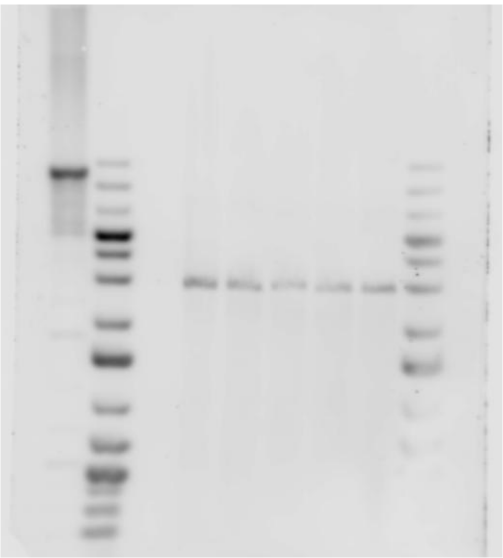

Figure S12 The full-length blot of Figure S4. The first land from left was supercoiled plasmid of pLS-HCas9-Mfa2

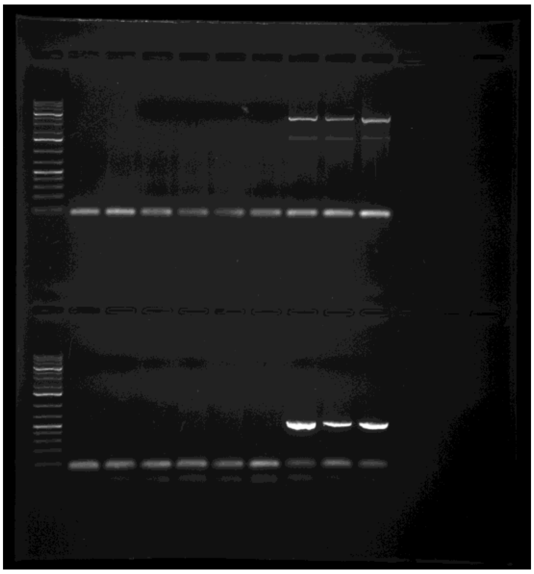

Figure S13 The full-length gel of Figure S6A &S6B.

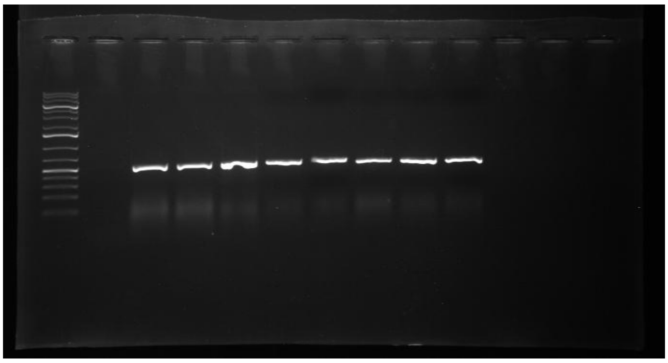

Figure S14 The full-length gel of Figure S6C.

S1 Tables

Table S1 Primers used in this work

Primer	Sequence
SsPu6F	5’-ATCGGCAGCAAAGGATACGATCGTCCCGACGATGCTC
SsPu6R	5’-TCTTCAGAGGTCTCTCGAGGGTAAAATCTGATTGTATGAG
U-F	5’-CTCCGTTTTACCTGTGGAATCG
gR-R	5’-CGGAGGAAAATTCCATCCAC
SsU6T mfa1①-	5’-AGGCCGAGGTCTGGGCGGTCGAGGGTAAAATCTGATTGTATG
gRTmfa1①+	5’-ACCGCCCAGACCTCGGCCTGTTTTAGAGCTAGAAAT
SsU6T mfa1②-	5’-CTCTTGCGGCCCTCATCGGCGAGGGTAAAATCTGATTGTATG
gRTmfa1②+	5’-CCGATGAGGGCCGCAAGAGGTTTTAGAGCTAGAAAT
SsU6T mfa2①-	5’-GGACGGAGGCAGCAACAGTCGAGGGTAAAATCTGATTGTATG
gRTmfa2①+	5’-ACTGTTGCTGCCTCCGTCCGTTTTAGAGCTAGAAAT
SsU6T mfa2②-	5’-CGGTGCAGTAGACTGCAGGCGAGGGTAAAATCTGATTGTATG
gRTmfa2②+	5’-CCTGCAGTCTACTGCACCGGTTTTAGAGCTAGAAAT
U-Fs-BamHI	5’-CTATGTTACTAGAGGATCCCGGAATGATCTACAAAGCGTTCTTC
gR-R-HindIII	5’-TAACCATGGTACCAAGCTTATTCCATCCACTCCAAGCTCTTG
Thph+	5’-ACCTGATGCAGCTCTCGGAGTTTTAGAGCTAGAAATAG
Thph-	5’-TCCGAGAGCTGCATCAGGTCGAGGGTAAAATCTGATTGTATG
ngU-FsBamHI	5’-CGACTCTAGAGGATCCCTTAAGCGGAATGATCTACAAAGCGTTCTTC
NGgR-RBamHI	5’-AATCACTAGGGGATCCATTCCATCCACTCCAAGCTCTTG
C mfa2 PstIF	5’-CTAAGCTTGCATGCCTGCAGGCTAGCCCATTGGGCACACCAG
new mfa2F	5’-GAAACTGTTGCTGCCTGTGTGCAAGCCATTGTTTCTGTTAACGAGC
new mfa2R	5’-TTGCACACAGGCAGCAACAGTTTCGAAGATGAACATGGTGAATTGGTAAA
C mfa2 PstIR	5’-CCTCTAGAGTCGACCTGCAGTTAGGCCACGGTGCAGTAGACTGC
mfa2C1F	5’-TGCCTGAATTGCTCCGCTTGTC
CasR01	5’-GGATACCGACCTTCCGCTTCTTC
mfa2C1R	5’-TGGCTCTGTTTCTCACGAGATCACG
HygR01	5’-TGTATGGAGCAGCAGACGCGCTAC
Gpd F	5’-GATTAGATCTTGCTGAT
Cmfa2R	5’-TTAGGCCACGGTGCAGTAGACTG
natR01	5’-CGGACTCCCGGACGTTCGTC
Hph F	5’-ATGAAAAAGCCTGAACTCACCGCG
hph1-F	5’-GCAAGACCTGCCTGAAACCG

hph1-R	5’-GGTCAAGACCAATGCGGAGC
cas9-F	5’-ATGGCTCCTAAGAAGAAGCGGAAGG
cas9-R	5’-TTACTTTTTCTTTTTTGCCTGGCCG
Pu6-F	5’-GCATGACGTTATTTATGAGGTGGG
sgRNA-R	5’-AAAAAAAGCACCGACTCGGTGCC
U6T 1621-	5’-GCTACTTCCTGCTGCGGACCGAGGGTAAAATCTGATTGTATG
RT 1621+	5’-GTCCGCAGCAGGAAGTAGCGTTTTAGAGCTAGAAATAG
U6T 6375-	5’-CATCGTGCCGCCTGCCCAGCGAGGGTAAAATCTGATTGTATG
gRT 6375+	5’-CTGGGCAGGCGGCACGATGGTTTTAGAGCTAGAAATAG
U6T 5775-	5’-GCGTCCAAGACCCTGGTCACGAGGGTAAAATCTGATTGTATG
gRT 5775+	5’-TGACCAGGGTCTTGGACGCGTTTTAGAGCTAGAAATAG
U6T 5019-	5’-CGCGAGATGCTCGCAGCCACGAGGGTAAAATCTGATTGTATG
gRT 5019+	5’-TGGCTGCGAGCATCTCGCGGTTTTAGAGCTAGAAATAG
U6T 3949-	5’-GGTCGCTTGGAGGGCGGAACGAGGGTAAAATCTGATTGTATG
1621 F	5’-ATGTCGAACGTCAACACATCTAC
1621 R	5’-TACACGAGGTTTGTCAG
3949 F	5’-ATGCGAGACCAAGCTACCACGG
3949 R	5’-CTTGCCCTGCGCCTTGAGAATG
5019 F	5’-CTCGCCTCTCAAGGATATTTCGG
5019 R	5’-TCCACAACCCAGTCTGCAGTGC
5775 F	5’-TACTGCAGACCATCTGACAGCCAG
5775 R	5’-TGGAGTCAACACACAGGGTCCC
6375 F	5’-GGTCGTAGCCCCTCGTTTCTTG
6375 R	5’-AATGCCCGTCTCGAACCACTCG
